# A Holistic Approach to the Patients/ Families with Inborn Errors of Metabolism

**DOI:** 10.34763/jmotherandchild.20202402si.2004.000010

**Published:** 2020-10-02

**Authors:** Peter Burgard

**Affiliations:** 1 Department of General Pediatrics, Division of Child Neurology and Metabolic Medicine, Center for Child and Adolescent Medicine, University Hospital Heidelberg, Heidelberg, Germany

**Keywords:** health care organization, integrative medicine, systems integration, inborn errors of metabolism

## Abstract

**Background:**

Diagnosis, treatment, and care of inborn errors of metabolism require well organized interdisciplinary teams. Holistic approaches comprise the system of all elements and relations between elements necessary for an optimal function of the system.

**Methods:**

Following the rule “structure follows function” based on scientific, academic, and clinical experience the elements of the system providing diagnosis, treatment, and care for inborn errors of metabolism are defined and described.

**Results:**

A holistic approach to inborn errors of metabolism comprising 10 elements is suggested, established, and controlled by an interdisciplinary metabolic team organized as a disease, and a case management program based on evidence-based guidelines is suggested. Quality assurance and quality control will not only improve the treatment of the individual but also the health system.

**Conclusion:**

The holistic approach is a joint project of the team of health care professionals and the person with a condition, allowing them to see the patient’s individual medical, behavioral, social, legal, and economic context. For practical, technical, and economic reasons this will only be possible in centers caring for a critical number of individuals.

## Keypoints

- The holistic approach describes the system in which diagnostic service, treatment, and care happens, given the economic, technical, personnel constraints of the health care delivering team as well as the individual background of the patient.
- The holistic approach is systematic, i.e. all elements are explicitly specified regarding their structure and function
- The explicit description allows adaptation and improvement of the system after evaluation by quality management and quality assurance linked to medical guidelines and public health policies

## Background

### Inborn errors of metabolism (IEMs)

For practical reasons, this article focuses on treatable IEMs, which does not exclude that untreatable IEMs can be approached from a holistic perspective. However, tertiary prevention, and palliative medicine in particular, are specialties by themselves that follow a holistic approach (1), and that is why individuals with untreatable IOMs should always be transferred to a palliative team.

Treatable IEMs represent many groups of disorders – each group containing several diagnoses – with a broad spectrum of clinical manifestations (acute vs. chronic onset of reversible or irreversible signs and symptoms), prognosis (from infaust to normal), and different pathways of inheritance, treatment, and outcome (2), where each group, and sometimes even each disorder needs special expertise. Besides, within each disorder, there can be interindividual genotypic and phenotypic variability from severe to potentially benign metabolic phenotypes (3). On the other hand, treatable IEMs share significant facets: (a) they are inherited, i.e. may cluster within families and are transmitted over generations with the probability of recurrence (b) they are chronic conditions, often associated with the necessity of lifelong day-to-day management, taking into consideration the general health status as well as growth and development, (c) management is most often multimodal, including diet, drugs, and skilled behavior, (d) management is executed by the family and/or the patients themselves, however, guided by an experienced metabolic team, (e) close and reliable mutual feedback between management and guidance is essential for effective and efficient monitoring of treatment and outcome.

### What is meant by “holistic?”

The word “ὅλος,” in Greek means “entire, the whole” (why sometimes the term wholistic is used), or “integrally”. An intuitively easier understanding can be gained from the perspective of general systems theory (4, 5), defining the structure of a system as a set of elements (parts) that are connected by relations. The elements can get inputs from outside of the system or from other elements within the system and can produce outputs. A system is called open when not all elements get inputs and produce outputs. In addition to the structure, systems can be described by their function. For example, looking at the urea cycle from a systems perspective, the structure consists of four biochemical compounds (ornithine, citrulline, argininosuccinate, and arginine) plus five core enzymes, one activating enzyme, and one antiporter, whereby the function is the detoxification of ammonia (input) to urea (output). The fact that elements plus relations define a system is why it is said that the whole is more than the sum of its parts (elements).

If the elements are active, i.e. influencing other elements, the system is called dynamic. A dynamic system that can maintain its structure and function is called to be in a steady-state, and it is stable if it can return to its initial state after a disturbance. Inputs and outputs can be material (like in the urea cycle), information (e.g. explaining the result of newborn screening to parents), or both (e.g. monitoring systems for inborn errors of metabolism (6) or transition programs from pediatrics to adult medicine (7).

So far it should become obvious that what is meant by the “whole” can and must be explicitly described, and that expressions like “everything is a system” or “everything is related to everything” are not very helpful, as they do not allow to distinguish different systems, but end up with one single system (what is called “the total approach” in contrast to the “holistic approach”). Systems should be described (and organized) in the most parsimonious way, which still allows adequate functioning. For example, when a laboratory result is communicated via the doctor to a patient, the information flow is transitive (i.e. if the lab sends a result to the doctor, and the doctor communicates the result to the patient, thenthe information from the lab is transmitted to the patient as the final receiver), but this will not mean that the lab is in direct contact with the patient.

There are also other concepts describing similar ideas. For example, in 2016, the Archive of Disease in Childhood published a series of articles (8, 9, 10, 11) dealing with the “integrative care model,” which avoids fragmentation between and within public services, by creating an “umbrella team,” making moves toward integration of infant, children, and young people’s health services (8). However, this model is a generic political and organizational framework focused on the UK health system, and it has to be translated to specific diagnoses (12).

### The holistic approach to families/patients with IEMs

The holistic structure and thereby the boundaries of a system is defined by its function or aim. The aim of the holistic approach to families/patients with IEMs is to provide the best evidence-based diagnostic service, treatment, and care in the most efficient way, given economic, technical, personnel constraints of the health care delivering team as well as the individual background of the patient, where treatment refers to what medical measures are possible and necessary to manage the disease of an individual patient, and care refers to how the treatment of the individual patient has to be managed, as well as additional para-medical interventions.

## Methods

Following the rule “structure follows function” based on the author’s scientific, academic, and clinical experience in the field of IEMs, the elements of the system providing diagnosis, treatment, and care are defined and described after a content analysis of each element (subsystem).

## Results

Table 1 defines a list of ten elements of the holistic approach, and it becomes obvious that each element also can be regarded as a system by its own, in other words, the holistic approach consists of a system of subsystems and the achievement of its function depends on how the relations (in computer language the interfaces) between the subsystems are organized. The suggested system does not exclude that some readers may see less or even more subsystems or will stress the importance to further subdivide single subsystems. Following the definition of the aim of our approach, for each element examples for diagnosis, treatment and care are given.

**Table 1:** Ten elements (subsystems) of a holistic approach to IEM

	Element/subsystem	Diagnosis	Treatment	Care
**1**	**Metabolic disorder**	Genes Enzymes Metabolites Organs (e.g. brain, liver, kidney) Outcomes	Diet Drugs Enzymes Genes Behavioural measures (e.g. eating at night, writing food protocols)	Metabolic monitoring Clinical monitoring
**2a**	**Patient**	Early, late onset subtypes Severity	What, how much, how long	Where, what
**2b**	**Person**	Understanding the condition	Application of treatment recommenda- tions in everyday life (compliance, adherence)	Coping with the condition in everyday life Support Social relations
**3**	**Family**	Understanding the condition Genetic counseling	Application of treatment recommendations in everyday life (compliance, adherence)	Technical, emotional, social, informational support Social relations
**4**	**Health Care Professionals**	Biochemistry	Pediatric and adult metabolic specialists Dietetics Nutritionists	Clinical medicine Psychology Social work Physiotherapy Occupational therapy Empowerment by professionals
**5**	**Patient advocacy groups**	Role models for coping and outcomes at different ages	Exchange of tips and tricks for everyday management	Social, informative, political support, Empowerment by peers
**6**	**Technical devices**	Laboratory methods	Monitoring systems	Aids and tools
**7**	**Science**	Etiology Pathomechanism Basic research	New treatments Clinical research on long-term outcome	Guidelines and standards Professional training
**8**	**Legal constraints**	Newborn screening programs Whole exome/genome sequencing	Regulations for reimbursement	Social legislation (e.g. inclusion of handicapped people, financial support) Transition politics
**9**	**Economic aspects**	Cost-effectiveness	Cost-effectiveness Additional costs for patients and families	Cost-effectiveness
**10**	**Industries**	Innovations of diagnostic technology	New treatments and products	Written and electronic information Sponsured conferences

### Content Analysis of the Ten Elements of the Holistic Approach

#### The metabolic disorder

IEMs are inherited disorders of metabolic pathways leading to non-physiological accumulation or depletion of metabolites. Diagnoses can be confirmed on different biochemical levels (genes, enzymes, and metabolites), offering specific treatment options (special diets, drugs, enzymes, genes, and behavioural measures like eating regularly calculated amounts of nutrients, even at night, writing food protocols, putting a nasogastric tube or operating a syringe pump) requiring biochemical as well as clinical monitoring. Diagnoses can be clustered into nosological categories (13), but each disorder has specific characteristics that must be addressed in a disorder-specific organization of necessary procedures for diagnosis, treatment, and care.

#### The individual as a person and as a patient

The individual with an IEM (in the same way as with any disease) enters the system with two roles, as a patient, and as a person. Although this is an analytical distinction, a patient is a person with a medical disorder, it is helpful to define the tasks but also the boundaries of a holistic approach to an IEM. Not every issue mentioned by a patient has to be handled by the metabolic team, in principles only those interfering with actions of diagnosis, treatment and care. After instruction and training within the tolerance of evidence-based recommendations, the management of treatment and monitoring is transferred to the patient system. Merging the roles of the patients and the persons will lead to a diffusion of the boundaries of the medical setting. Nevertheless, the health care team has to acknowledge, that embedding a twenty-four seven treatment in everyday life can be a significant personal challenge.

#### Patients are individuals in medical or paramedical contexts

In principle this is what case reports in scientific journals or case examples in textbooks communicate in the descriptions of individual patients, with the exception that patients’ files are identified, i.e. patients have a name, a birth date, a unique diagnostic pattern, and a unique history of the disease.

#### Persons are individuals in contexts outside medical contexts

The person-subsystem describes the individual in his/her daily life including personal values, preferences, attitudes, individual and social behaviors, rights, duties, and obligations. Parents of individuals with an IEM will not say they have a patient, instead, they will say they have a child, and in everyday life, the individual will not call himself/herself a patient but a person, however, a person with a condition (14). In a holistic approach, a subset of the attributes of the individual as a person will be related to the attributes of the individual as a patient. Ubiquitous examples in IEMs are patients’ compliance or adherence with recommendations and health-related quality of life. There is no general algorithm to determine the subset of personal attributes, but as a working hypothesis, the person subset can be described by all those attributes relevant for the diagnosis, treatment, and care of the metabolic disorder and the management of the patient. This subset can be different for any individual patient, why active listening in medical history taking is essential (15).

The bioecological model, originally proposed by Bronfenbrenner (16, 17, 18) to describe the individual child with his unique traits (genes, sex, age, temperament, health status, intelligence, etc.) in the center of different levels of environmental influences, is an excellent template for the concept of a person.

The model comprises four nested (i.e. hierarchical) system layers, comparable to a Russian Matrjoschka, showing a gradient from direct influences at the level of the microsystem to more and more indirect influences from the meso- via the exo- to the macrosystem. A fifth system, the chronosystem, is introduced to map individual and historical development. The five systems and examples of their elements can be summarized as follows:

The microsystem represents the immediate, i.e. personally experienced environment

- Family
- Peer group
- School
- Healthcare professionals

The mesosystem contains junctions/connections between different microsystems

- Supportive connections result in positive effects
- Non-supportive connections result in negative effects

The exosystem contains environments to which the individual does not personally belong to, but which may have effects on the individual

- Parental working conditions
- Institutions and legal regulations (school and health systems)
- Economic aspects
- Media

The marcosystem is the context in which all other systems are embedded: customs, conventions, values, laws, culture, and subculture (e.g. conceptions of child-rearing or interaction), and social stratification.

The chronosystem represents the historical change of values, technologies, and the overall social circumstances but also the chronological and developmental age of the individual, e.g. the emergence of the digital era or whole-exome sequencing, the change from paternalistic to cooperative medicine with the conception of the patient as an autonomous agent, the policy of transition and transfer from pediatric to adult medicine (7, 19).

### The family subsystem

In the field of IEMs, the family subsystem should be conceptualized as the extended genetic family. On the one hand, the family is a resource system, giving practical, technical, and emotional support to the patient but also informational support to health care professionals (HCPs), but a family member with an IEM may also be a risk for other family members, e.g. leading to emotional and financial parental stress (20, 21) or affecting healthy sibling’s mental health and psychosocial adjustment (22). On the other hand, inherited diseases create the need for genetic counseling. Roughly one index case with an autosomal recessively inherited trait may be associated with nine other people seeking genetic information (1 sibling of the case, 2 parents, 1 sibling of the parents and its spouse, 4 grandparents).

### Health care professionals (HCPs)

**Figure 1:**
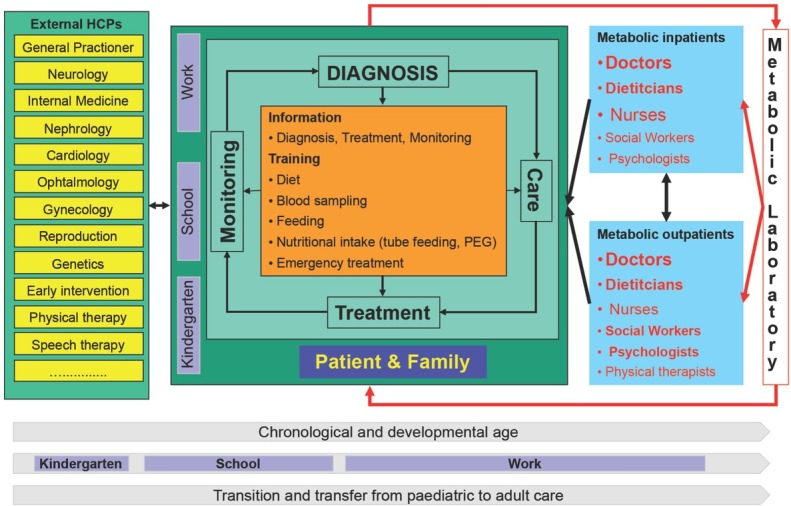
Prototypical team of healthcare professionals for patients with Inborn Metabolic Diseases.

Figure 1 shows a prototypical professional health care team for patients with an IEM. Teams for inpatients and outpatients in metabolic centers usually will constitute the primary point of care, but often external HCPs also consult. Not all medical and para-medical specialties will necessarily be involved with any IEM and depending on the specific diagnosis, severity of the genetic, biochemical, metabolic, and clinical condition of the patient the team will be larger or smaller. Professional teams can and should change with chronological and developmental age when new developmental tasks for the patients arrive. For most disorders, nursery-school and school teachers will be involved, at least informed about the basics of the condition. Programs of transition from pediatric to adult medicine may involve a transfer to a completely new professional team. However, for all IEMs a close and reliable loop from the clinical setting to the metabolic laboratory and back to the clinical setting is essential.

### Patient advocacy groups

Regional, national, or even international patient advocacy groups exist for nearly every IEM. They organize meetings in person and newsgroups and chatrooms on the internet, are represented in political institutions, and trigger research or medical guidelines

(23). Patients and parents can find new peer groups but also get role models for coping with different aspects of their condition at different ages or situations. HCPs should be aware that also patient organizations get sponsored from pharmaceutical industries potentially creating conflicts of interest (24).

### Technical devices

Metabolic medicine is impossible without hardware and software, partly extremely expensive and difficult to handle (amino acid analyzers, mass spectrometers, sequencers), partly low priced and easy to manage (food scales). Medical aids such as nasogastric tubes, syringe pumps, speech computers, or orthoses require individual adaptation and training, extending the team temporarily.

## Science

Evidence-based metabolic medicine by definition will result in a holistic approach and vice versa. Medical guidelines (25, 26, 27, 28) recommend what should be done, how, and by whom, thereby explicitly or implicitly following a systems or holistic approach (29). Though there is no doubt that medical interventions should be scientifically underpinned, this does not mean that every action has to be studied systematically. Many interventions can be justified on logical grounds. For example, the necessity of explaining the diagnosis and treatment to parents and patients or regular monitoring is obvious and will not require a randomized controlles trial (RCT) (30).

### Legal constraints

Like every approach to IEMs also the holistic approach is more or less regulated by laws (definition of screening panels, definition and equipment of reference centers, the legislation of genetic testing and counseling, abortion, transition politics, etc.), constituting constraints of the system as a whole. On the other hand, in most legislations individuals with chronic conditions have special rights (e.g. tax concessions, compensation for disadvantage, inclusion in school and society) however, only after individualized examination and allowance, so social workers are essential in a holistic approach. Scientific societies, but also patient organizations may also be involved in legislation.

### Economic aspects

There are many aspects regarding care for IEMs. Newborn screening panels are evaluated for cost-effectiveness (31), regulations regarding reimbursement of medical food and drugs are different between legislations sometimes leading to significant co-payment and loss of social and economic opportunities of patients and families (32). Finally, investment in a team of specialists only pays off for larger centers.

### Industry/Industries

In a series of publications in The Lancet (33, 34, 35), three roles of the industry have been formulated. The industry as an informant providing medical information (1), as a political player (2), and as a medicines provider (3). In fact, diagnosis, treatment, and care will not be possible without the industry. However, obligations to shareholders on the one hand and the community on the other may be imbalanced leading to efforts to mold medical thinking as a means to enlarge the market (36).

### Relations between the Elements of the Holistic Approach

Having defined the elements of the holistic approach, it remains to determine how these elements are related and how the system is controlled. From a formal point of view, 10 elements can have (10 × 9)/2 = 45 binary relations, leading to a system where every element is directly related to each other. Although this is true in principle, many relationships are indirect. Individual patients are members in patient advocacy groups, and these groups are political players in health legislation, but not every patient will be involved in this process. Biochemists are necessary to run the metabolic laboratory, but will not be in personal contact with the individual patient, scientists will transfer their results via HCPs to the patient. On the other hand, there may be unexpected direct relationships, e.g. between individual patients and the industry in cases of direct selling or direct-to-consumer advertising.

IEMs are very different, not only regarding the impairment of the metabolic pathway requiring different treatment, but also regarding the state of scientific research, clinical experience, and implementation into health systems (2). Besides, the phenotypic spectrum of diseases can be vast. Last, not least individuals with the same disease can be very diferent, more or less skilled to manage a diet or taking blood samples at home, anxious or optimistic, have a small unsupportive or a large supportive social network, be symptomatic or asymptomatic. Therefore, in the same way as treatment has to be individualized, also care and the holistic approach should be individualized. This leads to the question of who will establish, control and steer the system.

### Systems Management and Control

The team of HCPs described in Figure 1 is in the center of the holistic approach responsible for the management of the patient and the training and guidance of the person. The holistic approach is established by this team and optimally organized as a disease and case management program based on evidence-based guidelines, describing what has to be done as to why, how, when and by whom (25, 27, 37, 38). Such programs will also allow measures of quality assurance and quality control, improving not only the treatment of the individual but also the health system (29).

## Discussion

The management of an individual with an IEM is a joint project of the disease-specific team of HCPs and the individual as a patient and a person with variable contributions of both partners, depending on the disorder and the specific task. In everyday situations the condition is primarily managed by the person, however, guided by skilled, experienced, and organized metabolic teams, whereas in emergencies the patient is primarily managed directly by the specialist. The holistic approach allows us to see the patient in his or her individual medical, behavioral, social, legal, and economic context, but at the same time reveals how this context has to be organized and managed. For practical, technical, but also economic reasons this will only be possible in centers (39) caring for a critical number of individuals, otherwise, though the approach will be effective, it would not be efficient, nor cost-effective.

## References

[1] Noyes J, Pritchard A, Rees S, Hastings R, Jones K, Mason H (2014). Bridging the gap: transition from children’s to adult palliative care: final Report. Bangor University, United Kingdom, Together for Short Lives.

[2] Saudubray JM, Sedel F, Walter JH (2006). Clinical approach to treatable inborn metabolic diseases: an introduction. J Inherit Metab Dis.

[3] Garbade SF, Shen N, Himmelreich N, Haas D, Trefz FK, Hoff-mann GF (2019). Allelic phenotype values: a model for genotype-based phenotype prediction in phenylketonuria. Genet Med.

[4] Boulding KE (1956). General systems theory—the skeleton of science. Manage Sci.

[5] Von Bertalanfy L (1950). The theory of open systems in physics and biology. Science.

[6] Ten Hoedt AE, Hollak CE, Boelen CC, van der Herberg-van de Wetering NA, Ter Horst NM, Jonkers CF (2011). “MY PKU”: increasing self-management in patients with phenylketonuria. A randomized controlled trial. Orphanet J Rare Dis.

[7] Mazur A, Dembinski L, Schrier L, Hadjipanayis A, Michaud PA (2017). European Academy of Paediatric consensus statement on successful transition from paediatric to adult care for adolescents with chronic conditions. Acta Paediatr.

[8] Ewing CI, Cropper SA, Horsburgh TB (2016). Developing, implementing and evaluating integrated care models for infants, children, young people and their families. Arch Dis Child.

[9] Watson M (2017). The importance of engaging users and measuring outcome for integrated care: response to Ewing and Woodman. Arch Dis Child.

[10] Wolfe I, Lemer C, Cass H (2016). Integrated care: a solution for improving children’s health?. Arch Dis Child.

[11] Woodman J, Lewis H, Cheung R, Gilbert R, Wijlaars LP (2016). Integrating primary and secondary care for children and young people: sharing practice. Arch Dis Child.

[12] Bali A, Hargreaves DS, Cowman J, Lakhanpaul M, Dunkley C, Power M (2016). Integrated care for childhood epilepsy: ongoing challenges and lessons for other long-term conditions. Arch Dis Child.

[13] Ferreira CR, van Karnebeek CDM, Vockley J, Blau N (2019). A proposed nosology of inborn errors of metabolism. Genet Med.

[14] Cederbaum JA, LeMons C, Rosen M, Ahrens M, Vonachen S, Cederbaum SD (2001). Psychosocial issues and coping strategies in families affected by urea cycle disorders. J Pediatr.

[15] Forsyth RJ (2018). We have to talk about health-related quality of life. Arch Dis Child.

[16] Bronfenbrenner U, Morris PA (1998). The ecology of developmental processes. p. 993-1028. In: Damon W (editor in chief), Lerner RM (volume editor). Handbook of child psychology. Vol. 1: Theoretical models of human development. 5th ed.

[17] Bronfenbrenner U, Morris PA (2006). The bioecological model of human development. p. 793–828. In: Damon W (editor in chief), Lerner RM (volume editor). Handbook of child psychology. Vol. 1: Theoretical models of human development. 6th ed.

[18] Siegler R, DeLoache J, Eisenberg N (2014). How children develop. 4th ed.

[19] Grasemann C, Matar N, Bauer J, Manka E, Mundlos C, Krude H (2020). Ein strukturierter Versorgungs-pfad von der Pädiatrie in die Erwachsenenmedizin für Jugend-liche und junge Erwachsene mit einer seltenen Erkrankung. Monatsschr Kinderheilkd.

[20] Gramer G, Haege G, Glahn EM, Hoffmann GF, Lindner M, Burgard P (2014). Living with an inborn error of metabolism detected by newborn screening-parents’ perspectives on child development and impact on family life. J Inherit Metab Dis.

[21] Weber SL, Segal S, Packman W (2012). Inborn errors of metabolism: psychosocial challenges and proposed family systems model of intervention. Mol Genet Metab.

[22] Besier T, Hölling H, Schlack R, West C, Goldbeck L (2010). Impact of a family-oriented rehabilitation programme on behavioural and emotional problems in healthy siblings of chronically ill children. Child Care Health Dev.

[23] Hagedorn TS, van Berkel P, Hammerschmidt G, Lhotáková M, Saludes RP (2013). Requirements for a minimum standard of care for phenylketonuria: the patients’ perspective. Orphanet J Rare Dis.

[24] Evans I, Thornton H, Chalmers I, Glasziou P (2011). Testing treatments: better research for better healthcare. 2nd ed.

[25] Boy N, Mühlhausen C, Maier EM, Heringer J, Assmann B, Burgard P (2017). Additional individual contributors. Proposed recommendations for diagnosing and managing individuals with glutaric aciduria type I: second revision. J Inherit Metab Dis.

[26] Burgard P, Ullrich K, Ballhausen D, Hennermann JB, Hollak CEM, Langeveld M (2017). Issues with European guidelines for phenylketonuria. Lancet Diabetes Endocrinol.

[27] Häberle J, Boddaert N, Burlina A, Chakrapani A, Dixon M, Huemer M (2012). Suggested guidelines for the diagnosis and management of urea cycle disorders. Orphanet J Rare Dis.

[28] van Spronsen FJ, van Wegberg AM, Ahring K, Bélanger-Quintana A, Blau N, Bosch AM (2017). Key European guidelines for the diagnosis and management of patients with phenylketonuria. Lancet Diabetes Endocrinol.

[29] Heringer J, Boy SP, Ensenauer R, Assmann B, Zschocke J, Harting I (2010). Use of guidelines improves the neurological outcome in glutaric aciduria type I. Ann Neurol.

[30] Burgard P, Hoffmann GF, Zschocke J, Nyhan WL (2017). editors. Inherited metabolic diseases: a clinical approach. 2nd ed.

[31] Pfeil J, Listl S, Hoffmann GF, Kölker S, Lindner M, Burgard P (2013). Newborn screening by tandem mass spectrometry for glutaric aciduria type 1: a cost-effectiveness analysis. Orphanet J Rare Dis.

[32] Schieppati A, Henter JI, Daina E, Aperia A (2008). Why rare diseases are an important medical and social issue. Lancet.

[33] Abraham J (2002). The pharmaceutical industry as a political player. Lancet.

[34] Collier J, Iheanacho I (2002). The pharmaceutical industry as an informant. Lancet.

[35] Henry D, Lexchin J (2002). The pharmaceutical industry as a medicines provider. Lancet.

[36] Dukes MN (2002). Accountability of the pharmaceutical industry. Lancet.

[37] Baumgartner MR, Hörster F, Dionisi-Vici C, Haliloglu G, Karall D, Chapman KA (2014). Proposed guidelines for the diagnosis and management of methylmalonic and propionic acidemia. Orphanet J Rare Dis.

[38] Norris SL, Nichols PJ, Caspersen CJ, Glasgow RE, Engelgau MM, Jack L (2002). The effectiveness of disease and case management for people with diabetes. A systematic review. Am J Prev Med.

[39] Héon-Klin V (2017). European Reference networks for rare diseases: what is the conceptual framework?. Orphanet J Rare Dis.

